# The Co-N-C Catalyst Synthesized With a Hard-Template and Etching Method to Achieve Well-Dispersed Active Sites for Ethylbenzene Oxidation

**DOI:** 10.3389/fchem.2019.00426

**Published:** 2019-06-11

**Authors:** Chun Shen, Shanshan Jie, Hong Chen, Zhigang Liu

**Affiliations:** ^1^School of Chemistry and Chemical Engineering, Hunan University, Changsha, China; ^2^School of Materials Science Engineering, Foshan University, Foshan, China

**Keywords:** ionic liquids, Co-N-C, MgO, acid etching, hard-template

## Abstract

Biomass obtained from organic residues gradually becomes one of the optimal renewable feedstock of value added chemicals. Herein, the Co-N-C catalyst was prepared via a hard-template and etching method using the casein as C and N sources, magnesium oxide as the template, and cobalt porphyrin as the metal precursor. The obtained Co-N-C catalyst exhibited excellent catalytic performance for selective oxidation of ethylbenzene with a conversion rate of 96.5% under mild conditions. Moreover, the catalysts were investigated by techniques such as BET, XRD, Raman, transmission electron microscopic (TEM), and X-ray photoelectron spectroscopy (XPS). The results showed that the etching progress could improve the dispersion of Co and the exposure of active sites. Herein, the efficient oxidation of ethylbenzene was attributed to the well-dispersed Co-N species and the increased specific surface area.

## Introduction

The catalytic conversion of carbon feedstock like petroleum into high value chemicals has been of great importance in the chemical industry (Yang et al., 2015; Den et al., 2018; Pliekhov et al., 2018). And one of the most important chemical reactions in the chemical industry is the selective oxidation of aromatic hydrocarbon. Over the past decades, the precious-metal catalyst has accounted for the major share of the market of selective oxidation of aromatic hydrocarbon (Rosser et al., 2016; Wang et al., 2017). However, the ever-rising cost limited the industrial production and large-scale application of the precious metal catalysts (Chung et al., 2017; Wei et al., 2017). To date, an enormous amount of effort has been devoted to exploiting the noble-metal-free catalysts. Through long-term development, transition metals (Co, Fe, Ni), and nitrogen co-doped carbon materials (M-N-C) have broad application in the hydrogenation and oxidation reactions (Cheng et al., 2015; Liu et al., 2017; Jiang et al., 2018). Among them, a series of Co-N-C catalysts has been investigated in selective oxidation of aromatic hydrocarbon and exhibit excellent catalytic performance (Gutmann et al., 2013; Jie et al., 2017). At present, the high temperature pyrolysis method has been the main way to synthesize the Co-N-C catalysts. Generally, the dispersion and the size of active sites and the specific surface area are the key factor for catalytic performance. Nevertheless, Co species, the active sites of the catalyst, were more likely to aggregate during the pyrolysis, which had a great effect on the catalyst performance. In order to tackle the problem, it was of great necessity to synthesize a kind of Co-N-C catalyst with high dispersion clusters as active sites.

**Scheme 1:**
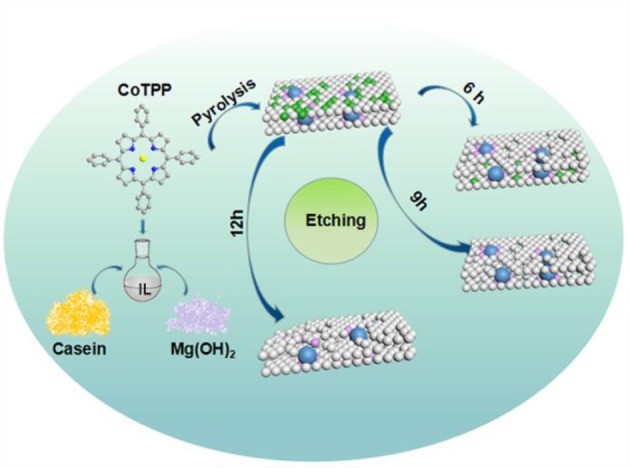
Schematic diagram of process to prepare M-Co-N-C-X catalysts.

Herein, cobalt porphyrin (CoTPP), 1-butyl-3-methyllimidazolium chloride and casein were chosen as metal, carbon and nitrogen precursors, and the magnesium hydroxide (Mg(OH)_2_) were introduced as the pore former (Wang et al., 2015). The detailed preparation process was shown in the Scheme 1. Generally, the 1-butyl-3-methyllimidazolium chloride was suitable to be used in the conversion of casein, which was rich in nitrogen, into porous carbon materials (Jia et al., 2017; Ding et al., 2019). The ionic liquids acted as a reaction medium and a porosity-directing regulator (Lee et al., 2010; Guo et al., 2013; Zhang et al., 2015c; Martinaiou et al., 2018). At the same time, Mg(OH)_2_ could be used to prevent the aggregation of metal particles and enlarge the specific surface areas when used as hard templates Liu W. G. et al., 2016; Liu X. B. et al., 2016. Furthermore, the unique Co-N_4_ coordination structure of the CoTPP could enhance the dispersion of metal nanoparticles and provide plenty of Co-N species (Jia et al., 2016; Chen et al., 2018). Thought the hard-template and etching method, the catalyst containing the highly dispersed active sites with nano-size was prepared and showed the excellent catalytic performance in the selective oxidation of ethylbenzene. In addition, the active sites of the catalysts and the effect of acid etching on catalytic activity were explored by BET, XRD, Raman, transmission electron microscopic (TEM), and X-ray photoelectron spectroscopy (XPS).

## Experimental

### Materials

Benzaldehyde, propanoic acid, pyrrole, dichloromethane (CH_2_Cl_2_), N, N-dimethyl-formamide (DMF), cobalt chloride hexahydrate (CoCl_2_·6H_2_O), ethanol, magnesium hydroxide (Mg(OH)_2_), 1-butyl-3-methyllimidazolium chloride (BmimCl), and casein were commercially available and used without further purification.

### Preparation of Cobalt (II) Porphyrin

The cobalt porphyrin was prepared as illustrated in the reported method. A mixture of benzaldehyde (0.05 mol, 5.6 g) and propanoic acid (180 mL) was added to 500 mL three-necked flask and heated to 130°C with stirring continuously, and then freshly distilled pyrrole (4.7 g) was added dropwise and kept the mixture refluxed for an hour. After the mixture cooled down to room temperature, 100 mL deionized water was inserted to the flask and then refrigerated overnight. The obtained precipitate was washed, filtered and purified by column chromatography to obtain the purple powder and denoted 5, 10, 15, 20-tetraphenylporphyrin (TPP). Subsequently, the TPP (1.0 g) was dissolved in 100 mL N, N-dimethyl-formamide (DMF) and heated to reflux, and then CoCl_2_·6H_2_O (2.5 g) was added until the porphyrin was exhausted. Following that, the production was diluted with deionized water (100 mL) and then refrigerated overnight. The product was washed with deionized water until the filtrate became neutral and then dried at 80°C for 12 h. The brown powder was obtained and denoted as CoTPP.

### Preparation of the Catalysts

The 1-butyl-3-methyllimidazolium chloride (BmimCl), casein and Mg(OH)_2_ were used to synthesize Co, N co-doped carbon materials, which served as carbon source, nitrogen source and hard template, respectively. In this typical synthetic procedure, BmimCl (2.0 g) was added to a 50 mL round flask and heated to 120°C with constantly stirring. And then, casein (0.4 g) was dissolved into the ionic liquid and obtained a homogeneous solution. Subsequently, CoTPP (0.1 g) and Mg(OH)_2_ (0.4 g) were mixed and then dispersed in the solution. The black viscous liquid was transferred to a quartz boat and then heated to 500°C in nitrogen atmosphere at a ramp rate of 5°C/min. The tube furnace was insulated for 2 h at this temperature, and the obtained powder was labeled as M-Co-N-C. The sample treated with 2 mol/L HCl solution for different length of time was denoted as M-Co-N-C-X (X means the acid treatment time). Similar to the above progress, the catalyst prepared without Mg(OH)_2_ or CoTPP was denoted as Co-N-C-X or M-N-C-X respectively. Beyond that, the M-Co-N-C-9 was recovered via readily centrifugation and then reused for several times to test the reusability and durability. The catalyst recycled for 5 times was donated as M-Co-N-C-9-R.

### Catalyst Characterization

Nitrogen adsorption-desorption isotherms were measured on Nova 1000e apparatus from Quanta Chrome Instruments at 77 K. The samples were outgassed at 200°C for 3 h prior to the measurements. In the relative pressure ranging from 0.05 to 0.98, the specific surface areas (S_*BET*_), the mesoporous volume (V_*total*_) and the pore size distribution (D_*P*_) were calculated using the Brunauer-Emmett-Teller (BET) and the Barrett-Joyner-Halenda (BJH) formula. Raman was conducted on Mono Vista 2560 Spectrometer with a 532 nm (2.33 eV) laser. High-resolution transmission electron microscope (HRTEM, JEOL-2100F) operating at 200 kV was carried to measure the morphology of samples. The mapping was conducted to determine the local elemental composition. X-ray photoelectron spectroscopy (XPS) was measured on a PHI 5000 CESCA system (Perkin Elmer) using Al Kα radiation (1486.6 eV). The X-ray diffraction (XRD) analysis was performed on a Japan XRD-6100 analyzer using Ni-filtered Cu Kα radiation with a scanning angle (2θ) ranging from 10°-80°, operated at 50 kV and 10 mA. The mass spectrometry analysis was performed on GCMS-QP2010ultra.

### Catalyst Test

To investigate the catalytic performance of the catalysts for selective oxidation of alkanes, the selective oxidation of ethylbenzene with TBHP as oxidant at 80°C was conducted. Before the reaction test, the catalyst (15 mg) and substrate (1.0 mmol) were put into the reaction tube. Then deionized water (3 mL) and TBHP (3.5 mmol, 70 wt% in water) were added sequentially. The mixture was heated to 80°C with magnetic stirring and kept for 12 h. After the reaction, ethyl acetate (9 mL) was added into the mixture to extract the filtrate and n-dodecane (100 μL) was added to the system as an internal standard. The obtained sample was quantitatively analyzed by using GC analysis. The catalyst was recovered, washed with ethanol and then dried in vacuum at 80°C.

## Results and Discussion

### Nitrogen Adsorption/Desorption Isotherms

**Figure 1:**
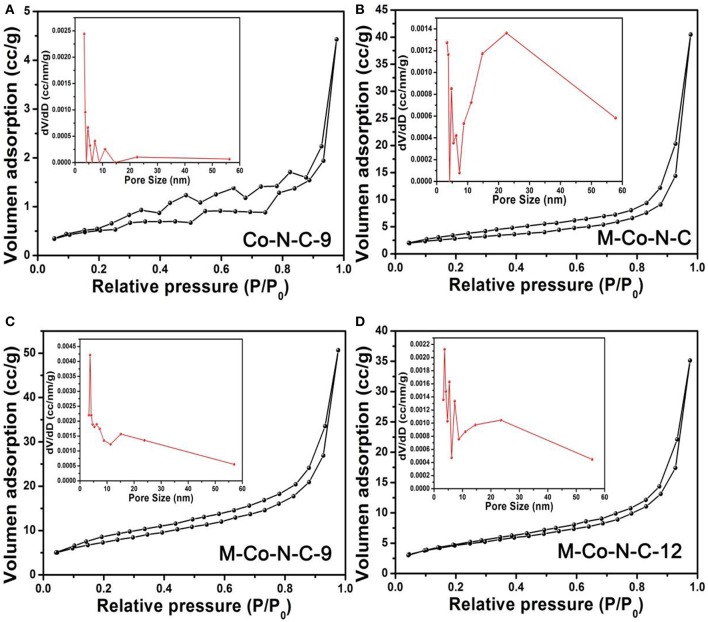
N_2_ adsorption-desorption isotherms of the **(A)** Co-N-C-9, **(B)** M-Co-N-C, **(C)** M-Co-N-C-9, and **(D)** M-Co-N-C-12.

N_2_ adsorption-desorption measurements operating at 77 K were performed to detect the samples. According to the Figure 1 and Figure S1, the N_2_ adsorption-desorption isotherm and pore size distribution of Co-N-C-9, M-Co-N-C, and M-Co-N-C-X revealed the pore textural properties. The isotherm of the samples showed a relatively slow increase in the range of 0.05 < *P*/P_0_ <0.80, and remarkable growth after *P*/*P*_0_ > 0.80, which indicated the N_2_ adsorption-desorption isotherms were attributed as type II curves (Thommes et al., 2015). As shown in the Figure 1, it was evidently clear that the hard template and etching progress were successful to form the pore structure. From the pore size distribution obtained from the Barrett-Joyner-Halenda (BJH) method, it was found that the M-Co-N-C-9 had the concentrated distribution of mesopores structure. The detailed information of the specific surface area and total pore volume of the samples were displayed in Table 1. It was noted that the specific surface area and total pore volume of M-Co-N-C-X grew gradually with the etching time increasing, and those of M-Co-N-C-9 were larger than other samples. As a general rule, enlarging the specific surface area of catalyst was conducive to exposing more active sites and strengthening the diffusion of substrates into the reaction regions, which might improve the catalytic performance of the samples (Zhang et al., 2015b; Wan et al., 2016; Wu et al., 2019). However, when the etching time was longer than 9 h, the specific surface areas and total pore volumes of the M-Co-N-C-12 decreased and it possessed more complicated pore size distribution, which might be on account of the damage of mesoporous structure.

**Table 1:** BET results of different catalysts.

**Sample**	***S_*BET*_*^a^ (m^**2**^/g)**	***V_*total*_*^b^ (cc/g)**	**$D_{\text{p}}^{\text{c}}$ (nm)**
Co-N-C-9	2.04	0.007	13.45
M-N-C-9	15.03	0.059	15.80
M-Co-N-C	6.87	0.034	15.13
M-Co-N-C-3	20.07	0.062	3.68
M-Co-N-C-6	22.40	0.065	3.73
M-Co-N-C-9	24.45	0.073	4.13
M-Co-N-C-12	16.53	0.054	3.73

a *Surface areas were calculated with the BET method, when the linear BET range was located at the P/P_0_ of ~0.05–0.30 for type II isotherm*.

b *Total pore volumes were estimated from the N_2_ adsorption isotherm at p/p_0_ = 0.98*.

*^c^ Mesopore diameter were calculated with the BJH method from the N_2_ desorption branch of the isotherm*.

### X-Ray Diffraction (XRD) Patterns Analysis

**Figure 2:**
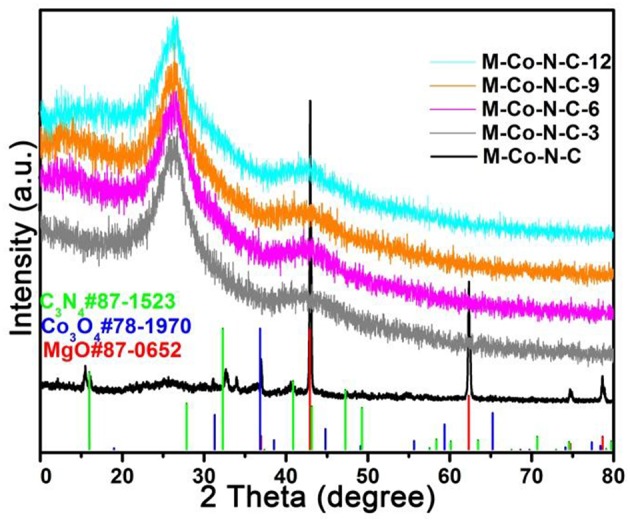
XRD patterns of M-Co-N-C, M-Co-N-C-3, M-Co-N-C-6, M-Co-N-C-9, and M-Co-N-C-12.

The crystalline structure of the M-Co-N-C-X (X = 3, 6, 9 and 12) was investigated by XRD. As shown in Figure 2, the diffraction peaks of M-Co-N-C at 36.9°, 42.8°, 62.2°, 74.5°, and 78.4° were ascribed to the characteristic (111), (200), (220), (311), and (222) reflections of MgO (JCPDS card No. 87-0652), respectively. And the weak peaks at 31.3°, 36.8°, 74.1°, and 78.4° were observed, which could be indexed to the crystalline facets (220), (311), (620), and (622) of Co_3_O_4_ (JCPDS card No. 78-1970), respectively (Chen et al., 2016). However, the catalytic performance of M-Co-N-C possessing MgO and Co_3_O_4_ was relatively low by comparing with M-Co-N-C-X, indicating that the MgO and Co_3_O_4_ were not the active species. Furthermore, the diffraction peaks at 16.0°, 32.3°, 43.1° and 74.5° can be attributed to the C_3_N_4_ (JCPDS card No. 87-1523), which suggested that the N atoms was doped into the carbon skeleton. Nevertheless, it was hard to detect any diffraction peaks in the samples treated with HCl, which might be ascribe to the fact that the etching progress removed the MgO and Co_3_O_4_, and destroyed the carbon structure (Zhang et al., 2015a).

### Raman Spectroscopy Analysis

**Figure 3:**
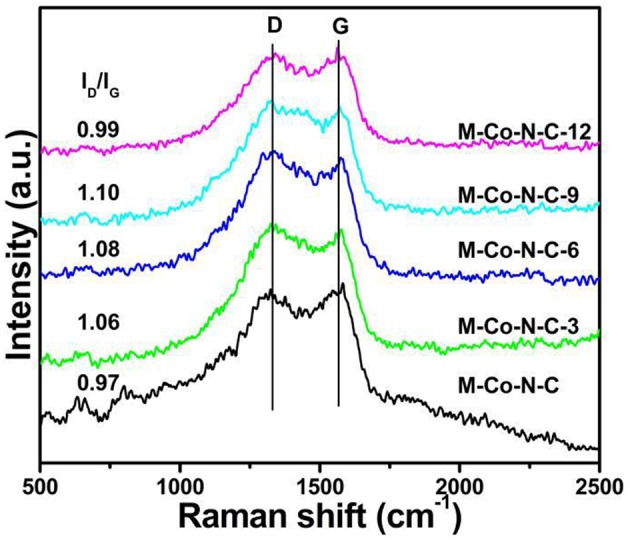
Raman spectra of M-Co-N-C, M-Co-N-C-3, M-Co-N-C-6, M-Co-N-C-9, and M-Co-N-C-12.

Raman spectroscopy was selected to detect the graphitization process and structural defects of the catalysts (Figure 3). An obvious D band at ~1,338 cm^−1^ associated with structure defects and a G band at ~1,572 cm^−1^ corresponding to graphitic carbon were obtained in the Raman spectroscopy (Guo et al., 2015; Yang et al., 2016b). Compared with the M-Co-N-C, the value of I_D_/I_G_ of the M-Co-N-C-X was higher. Moreover, the etching progress between carbon atoms and metal particles produced more defects with properly extending the time, which would promote the catalyst performance (He et al., 2017; Lin et al., 2018). In addition, it could be found that the D bands in M-Co-N-C-3, M-Co-N-C-6, and M-Co-N-C-9 were broader than M-Co-N-C and M-Co-N-C-12, suggested that the content of the doped N atoms in M-Co-N-C-3, M-Co-N-C-6, and M-Co-N-C-9 was higher than it in M-Co-N-C and M-Co-N-C-12. It might because the N atoms could change the electronic structure of the graphitic networks (Song et al., 2016).

### Transmission Electron Microscopic (TEM) Analysis

**Figure 4:**
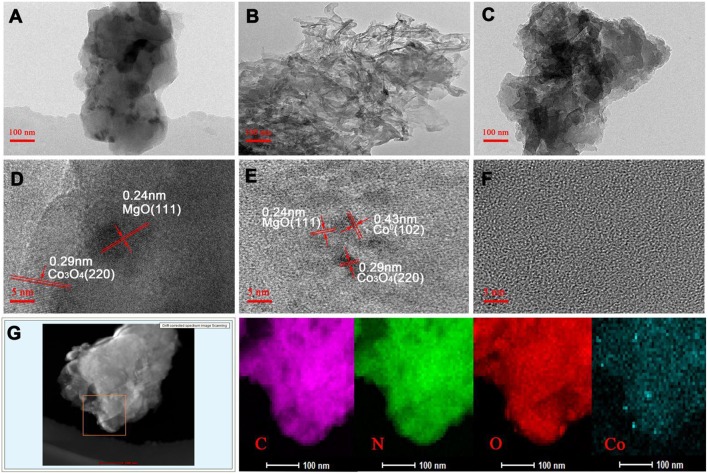
TEM images and HRTEM images of M-Co-N-C **(A,D)**, M-Co-N-C-6 **(B,E)**, M-Co-N-C-9 **(C,F)**, and elemental mapping of M-Co-N-C-6 **(G)**.

The morphology and structure of the M-Co-N-C-X composites were examined by TEM, high resolution transmission electron microscopy (HRTEM) and the elemental mapping (Figure 4). As seen from the HRTEM and TEM images, the number and diameter of nanoparticles on the carbon layer gradually decreased as the etching time increased. When the etching time was 9 h, no metal nanoparticles could be detected, which was because the etching reaction could effectively remove large metal particles such as MgO, Co_3_O_4_, and so on. Compared with MgO, the special structure of cobalt porphyrins, in which the Co atoms were strongly coordinated with N atoms, can effectively immobilize the cobalt atoms on the carbon layer, thus the Co species can be retained. As shown in Figures 4D,E, the HRTEM images of M-Co-N-C and M-Co-N-C-6 revealed the lattice fringe space value of 0.24 and 0.29 nm, which might be in good agreement with (111) facet of MgO and (220) facet of crystalline Co_3_O_4_ (Jin et al., 2015; Yan et al., 2016; Yang et al., 2016a; Wang et al., 2018). The result was in good agreement with the XRD analysis. Furthermore, it could also find the (102) facet of Co^0^ crystal with the d-spacing values of 0.43 nm in Figure 4E could also be found, but no nanoparticle in Figure 4F. Obviously, the large cobalt nanoparticles were etched and the remnants of cobalt particles were hard to be detected. In addition, elemental mapping analysis indicated that the existence of cobalt species and the uniform distribution of Co and N in M-Co-N-C-6, and revealed that the cobalt species have smaller scale, thus the effect of etching was confirmed. The high dispersion of active sites would be beneficial to the catalytic performance.

### X-Ray Photoelectron Spectroscopy (XPS) Analysis

**Figure 5:**
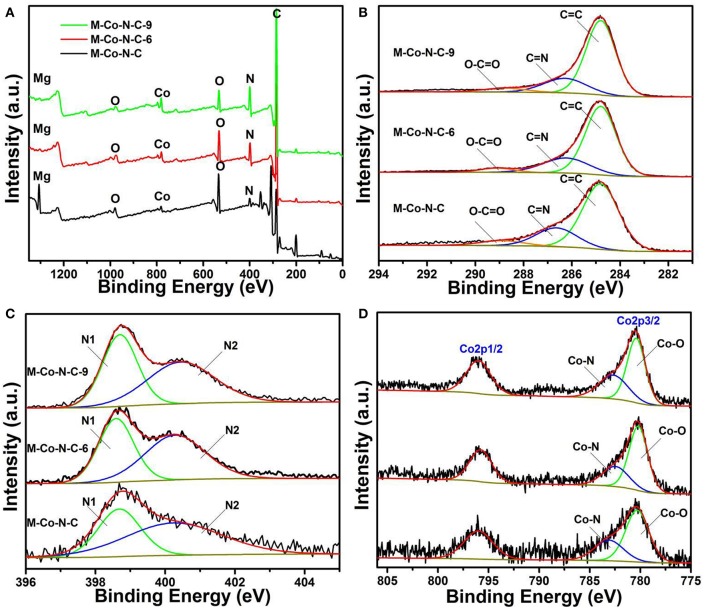
XPS spectra of as-prepared catalysts. **(A)** Survey, **(B)** C 1s, **(C)** N 1s and **(D)** Co 2p spectra of M-Co-N-C, M-Co-N-C-6, and M-Co-N-C-9.

X-ray photoelectron spectroscopy (XPS) was used to further explore the surface chemical composition of M-Co-N-C-X. As shown in Figure 5, the elements of Co, C, N, O, and Mg could be found in the full survey spectra of M-Co-N-C-X, which also revealed the relative contents of these elements. As shown in the Figure 5A, the full survey spectra of M-Co-N-C-X revealed the existence of C, N, O, Co, and Mg, indicated the trend of different elements with the etching time increasing. XPS survey spectra revealed that the content of Mg and O dropped rapidly, while the Co and N grew gradually when the sample was treated with more processing time. From the high-resolution C 1s spectrum (Figure 5B), three peaks at the binding energies of 284.8, 286.6, and 288.6 eV were assigned to C = C, C-N and O-C = O, respectively (Su et al., 2014; Lin et al., 2016). The N 1s XPS spectrum (Figure 5C) could be deconvoluted into two peaks: pyridinic N (N1, 398.6 eV), pyrrolic N (N2, 400.5 eV), respectively (Wu et al., 2014; Fu et al., 2015; Sun et al., 2017). And the Figure 5D presented the Co 2p XPS spectrum, where there were two broad binding energy peaks located at 779.5 and 795.5 eV corresponding to the Co 2p3/2 and Co 2p1/2 binding energy, respectively (Chao et al., 2014; Aijaz et al., 2015; Wu et al., 2017). The high-resolution of Co 2p3/2 could be fitted into Co-O (780.3 eV) and Co-N (782.6 eV). It was worth noting that it might be that Co-O or Co-N played a decisive role in in the reaction process.

Among the element composition of the M-Co-N-C and M-Co-N-C-6 as displayed in Table 2, it could be found that the content of O and Mg got a tremendous decline (54 and 99%), and the content of Co also had the obvious decline (16.5%), which indicated that the magnesium species and cobalt species, especially for magnesium oxide and cobalt oxides, were easily removed. When the etching time was more than 6 h, the content of Mg in the samples remained substantially unchanged, indicating the Mg atom has been basically cleared. But, comparing M-Co-N-C-6 and M-Co-N-C-9, the content of Co and N increased significantly (35 and 33%), which may be because that the carbon layer was destroyed when the etching time over 6 h and large numbers of Co, N were exposed. As literatures illustrated, the element of N, acting as ligands, and metal ions may form the complex Co-N_x_, generated excellent catalytic performance for the reaction (Puello-Polo and Brito, 2007; Datsyuk et al., 2008; Wei et al., 2015; Chen et al., 2017). The content of Co-N progressively increased with the etching time increasing from 0 to 9 h, but the content of Co-O reduced. It might be ascribed to the structure of Co-N was more stable than Co-O under the etching progress. When the etching time arrived 12 h, the content of the Co-N decreased which was attributed to the destroyed of the structure of Co-N.

**Table 2:** Elemental composition on the samples.

**Catalyst**	**C(at%)**	**O(at%)**	**Mg(at%)**	**Co2P(at%)**		**N(at%)**		
				**Total**	**Co-N**	**Total**	**N1 (398.6)**	**N2 (400.3)**
M-Co-N-C	57.7	16.5	18.92	1.33	17.9	5.63	39.0	61.0
M-Co-N-C-6	84.0	7.5	0.25	1.11	24.7	7.16	45.4	54.6
M-Co-N-C-9	83.6	5.14	0.25	1.50	25.8	9.50	47.1	52.9
M-Co-N-C-12	81.28	9.33	0.30	1.22	21.7	7.87	46.2	53.8

### Catalytic Performance of M-Co-N-C-X

The catalytic performance of the as-prepared catalysts was tested in selective oxidation of ethylbenzene with TBHP as oxidant and water as solvent, because the efficient oxidation of ethylbenzene account for a highly important position in the chemical industry. The analytic conditions of GC and gas chromatogram of selective oxidation of ethylbenzene using M-Co-N-C-9 as catalyst were shown in Figure S4, and the detailed data of all samples were listed in Table 3. Obviously, the catalytic activity of the sample prepared without CoTPP (M-C-9) was well controlled with only 62.8% of ethylbenzene conversion (Table 3, entry 1). Comparing M-C-9 and M-N-C-9 (Table 3, entry 1 and 2), the catalyst prepared via pyrolyzing porphyrin (TPP), casein and Mg(OH)_2_ presented relative high catalytic activity, suggesting that the doped of N atoms took an important part in promoting the reaction. Remarkably, the M-Co-N-C-9 showed more excellent catalytic performance than M-N-C-9 (Table 3, entry 7 and 2), which illustrated that the Co atom was also critical to catalytic performance. However, the activity of Co-N-C-9 without utilizing Mg(OH)_2_ as pore former sharply decreased (Table 3, entry 3). And combined with the result of BET analysis (shown in Table 1), it could deduce that the higher specific surface area and total pore volume could improve the catalytic performance. To thoroughly investigate the influence of the etching, the catalysts (M-Co-N-C-X) were used to perform the same experiments (Table 3, entry 4-8). Interestingly enough, the catalytic performance of the samples first increased and then decreased with the increase of etching time, but the M-Co-N-C-0 was the worst which indicated that the Co_3_O_4_ (detected in Figure 2 of XRD and Figure 4D of TEM) was not the active species. In contrast, the M-Co-N-C-9 had the optimum performance. According to the XPS analysis (shown in Table 2), the variation regularity of the catalytic performance of M-Co-N-C-X was in line with the content of Co-N, indicating that the Co-N species were the active sites. Combined with the above-mentioned characterizations and the experiments, it proved that the etching progress could expose more Co-N active sites which exerted decisive catalytic effect for the selective oxidation of ethylbenzene (Zhang et al., 2017).

**Table 3:** Catalytic performance of the catalysts for ethylbenzene oxidation.

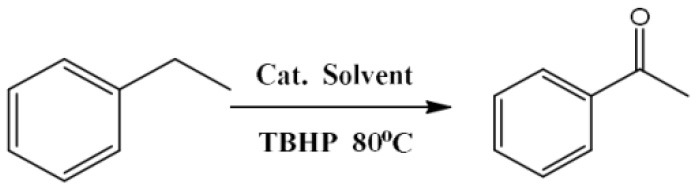			
**Entry**	**Catalyst**	**Conv./%**	**Sel./%**
1	M-C-9	62.8	91.8
2	M-N-C-9	82.8	94.6
3	Co-N-C-9	72.2	80.3
4	M-Co-N-C-0	75.7	84.2
5	M-Co-N-C-3	89.7	90.0
6	M-Co-N-C-6	93.3	94.2
7	M-Co-N-C-9	96.5	97.3
8	M-Co-N-C-12	92.6	88.5
9	M-Co-N-C-R	60.0	87.2

*Reaction conditions: ethylbenzene (1.0 mmol), TBHP (3.5 mmol, 70 wt% in water), catalyst (15 mg), 80°C; the conversion was determined by GC*.

### Applicability to Other Arylalkanes

The heterogeneous catalyst of M-Co-N-C-9 has been used to investigate the catalytic oxidation with a wide series of organic compounds containing C-H bonds with TBHP under similar conditions. The catalytic performance of the catalyst on these substrates displayed excellent results and some of the rules have been summed up. As shown in Table S1 (entries 2–4), the α-C connected with electrondonating or electron-withdrawing functional groups showed great activity during the catalytic oxidation process. In addition, for the p-substituted derivatives of ethylbenzene, the substituent played a decisive role in the reaction and displayed excellent conversion and selectivity to the corresponding ketones (Table S1, entries 5–6). It was amazing that, the selectivity of corresponding ketones of substrates containing two benzene rings reached up to 99%. In contrast, the catalytic activity for catalytic oxidation of naphthenic hydrocarbons was more moderate, because the C-H of cyclanes was inactive.

## Conclusions

In summary, the noble-metal-free catalyst of M-Co-N-C-9 with extremely tiny metal particles has been successfully prepared through an effective hard-template and etching method. A high catalytic activity over M-Co-N-C-9 with 96.5% conversion of ethylbenzene and 97.3% selectivity to acetophenone was observed for oxidation of ethylbenzene with TBHP as oxidant under the mild conditions, which may explain that the doping of ionic liquid and the etching of MgO made a critical contribution to form the unique defective structure and enlarge the specific surface area, so that massive active sites were exposed to the reaction substrate. According to a series of characterizations, the Co-N species were considered to be the vital active sites for enhancing the catalytic performance.

## Data Availability

All datasets generated for this study are included in the manuscript and/or the Supplementary Files.

## Author Contributions

CS and SJ designed and finished the synthesis and characterization analysis of materials and wrote the research paper. ZL and HC supervised the project, helped design the experiments, evaluated the data, and wrote the manuscript. The results of the manuscript were discussed by all authors.

### Conflict of Interest Statement

The authors declare that the research was conducted in the absence of any commercial or financial relationships that could be construed as a potential conflict of interest.
