# Supplementary material for: The Co-N-C Catalyst Synthesized With a Hard-Template and Etching Method to Achieve Well-Dispersed Active Sites for Ethylbenzene Oxidation
Source: Front Chem. 2019 Jun 11;7:426. doi: 10.3389/fchem.2019.00426 (PMC6580930; doi:10.3389/fchem.2019.00426)
Supplement: Supplementary file 1 [file Data_Sheet_1.docx]

Supplementary Material

The Co-N-C catalyst synthesized with a hard-template and etching method to achieve well-dispersed active sites for ethylbenzene oxidation

Chun Shen,^a^ Shanshan Jie,^a^ Hong Chen,^b^ Zhigang Liu ^a ,*^

^a^ School of Chemistry and Chemical Engineering, Hunan University, Changsha, Hunan, 410082, China

^b^ School of Materials Science Engineering, Foshan University, Foshan, Guangdong, 528000, China

*** Correspondence:**Zhigang Liu
[liuzhigang@hnu.edu.cn](mailto:liuzhigang@hnu.edu.cn)


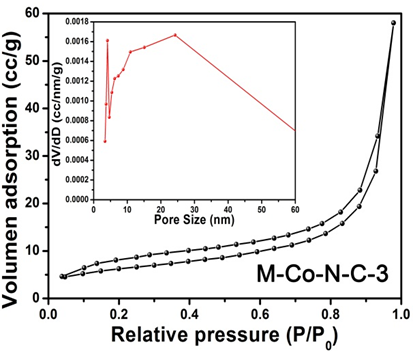

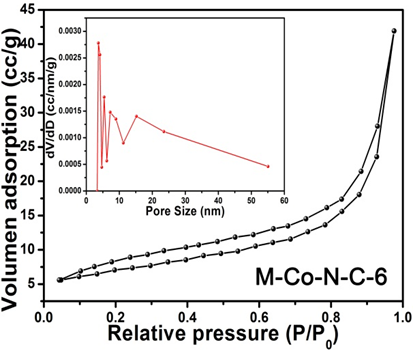


**FIGURE S1 |** N_2_ adsorption-desorption isotherms of the M-Co-N-C-3 and M-Co-N-C-6


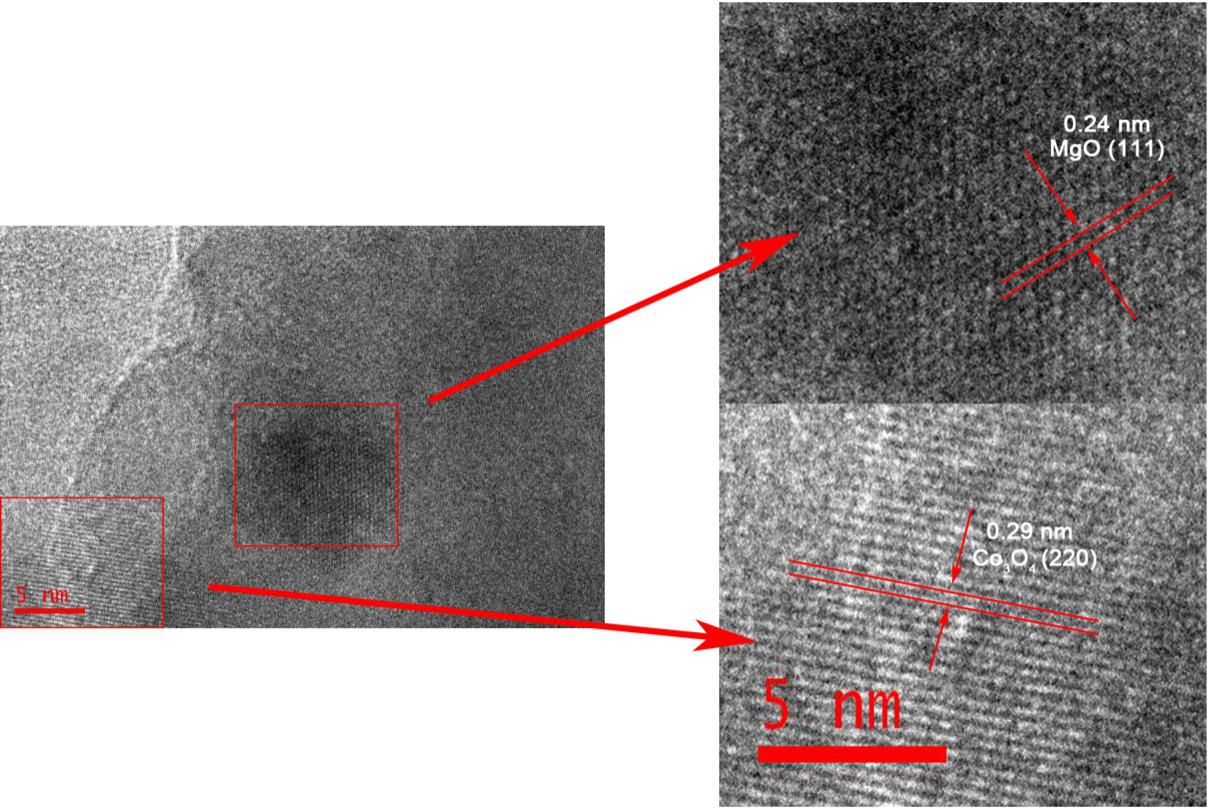


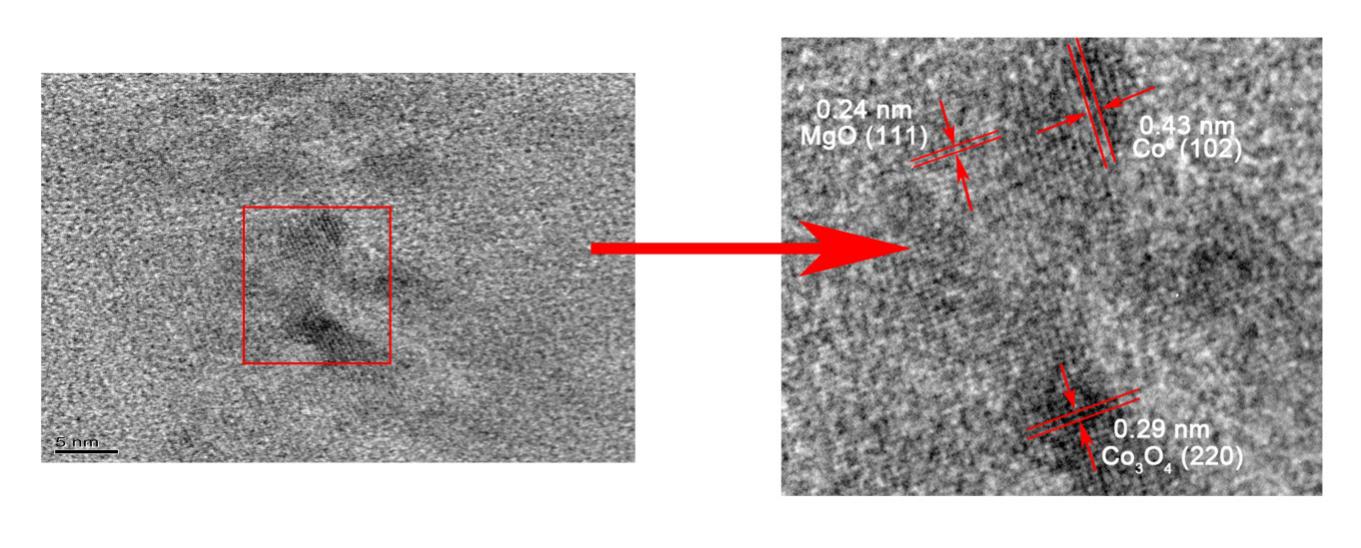


**FIGURE S2 |** Enlarged drawing of HRTEM images of M-Co-N-C and M-Co-N-C-6

**
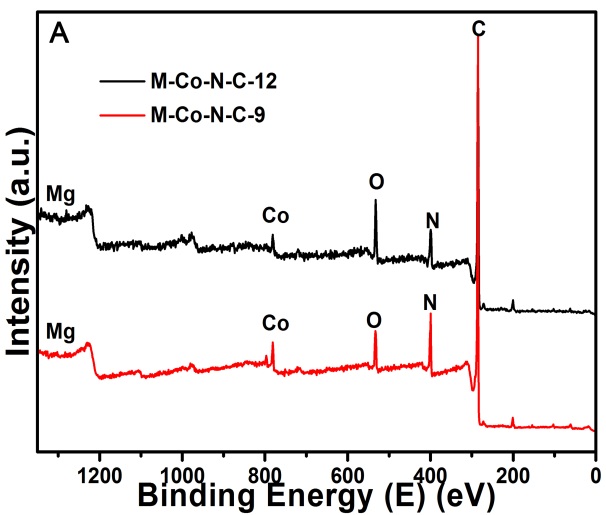

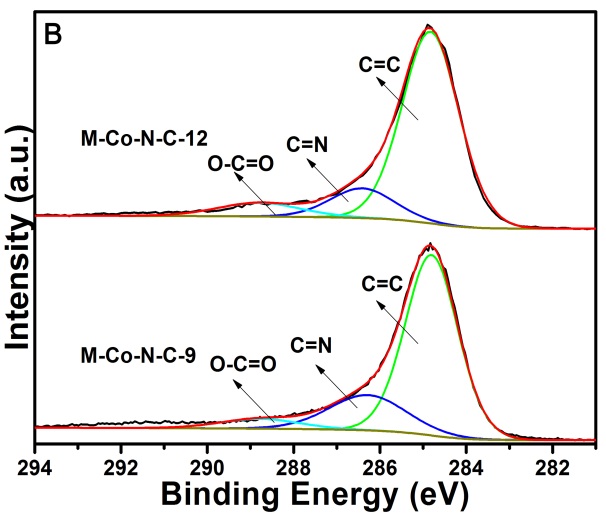
**
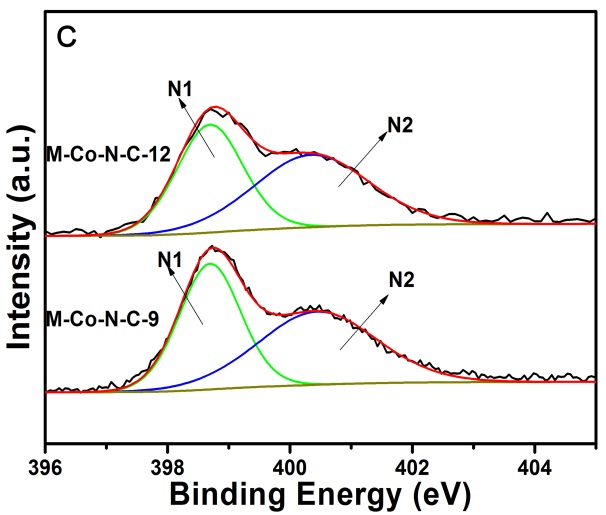

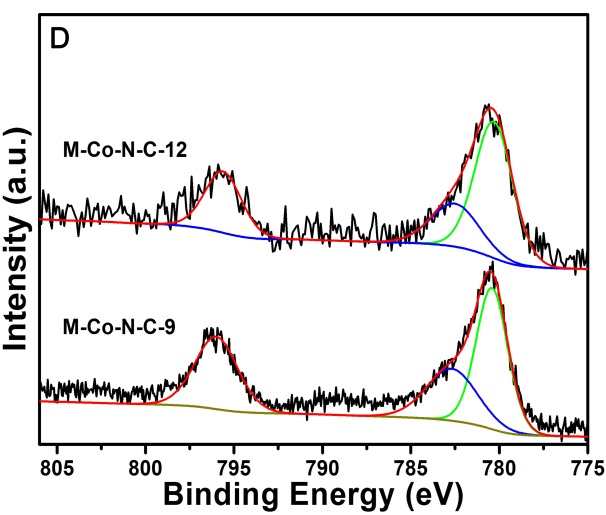


**FIGURE S3 |** XPS spectra of M-Co-N-C-9 and M-Co-N-C-12, (A) Survey, (B) C 1s, (C) N 1s and (D) Co 2p.


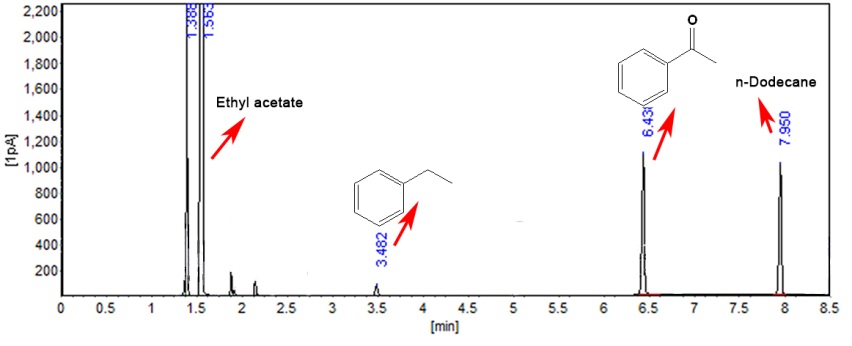


**FIGURE S4 |** Gas chromatogram of selective oxidation of ethylbenzene using M-Co-N-C-9 as catalyst.

Analytic conditions of GC (A90 ECHROM)：

High-purity hydrogen is used as the combustion gas; high-purity air is used as the combustion gas; and high-purity nitrogen is used as the carrier gas and the makeup gas.

Chromatographic column: GSBP-5

Detector: flame ionization detector (FID)

Injection temperature: 230 ^o^C Detector temperature: 230 ^o^C

Column temperature: keeping the temperature at 60 ^o^C for 3 min; the temperature rises at the rate of 15 ^o^C / min to 160 ^o^C, keeps for 6 min; the temperature rises at the rate of 24 ^o^C / min to 200 ^o^C, keeps for 15 min.

Hydrogen flow rate: 30 mL / min

Air flow rate: 300 mL / min

Nitrogen flow rate: 20 mL/min

Injection volume: 0.4 μL Collection time: 8.5 min

Internal standard: n-dodecane

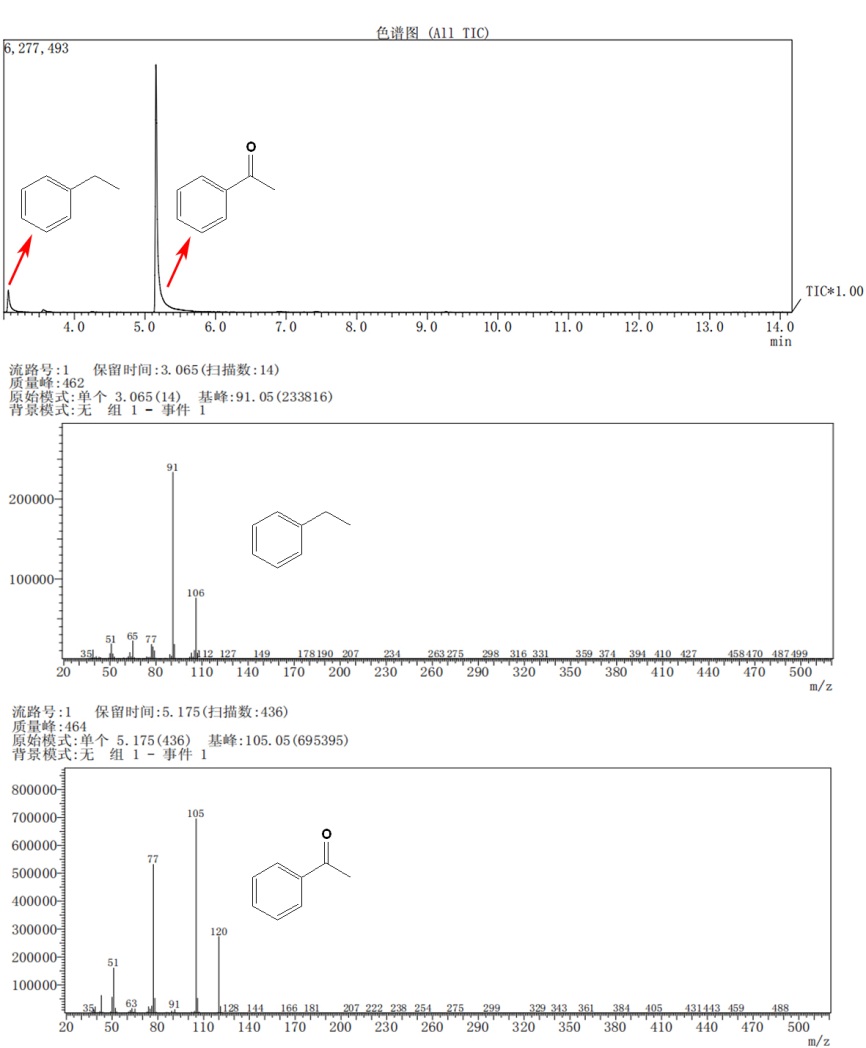


**FIGURE S5 |** Gas chromatography and mass spectroscopy (GCMS) analysis of selective oxidation of ethylbenzene using M-Co-N-C-9 as catalyst.

The GCMS analysis showed that the main product of selective oxidation of ethylbenzene using M-Co-N-C-9 as catalyst was the acetophenone.

| **Table S1 \|** Catalytic oxidation several of substrates by M-Co-N-C-9. | | | | |
| --- | --- | --- | --- | --- |
| Entry | *Substrate* | *Product* | *Conv./%* | *Sel./%* |
| 1 | 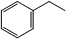 | 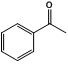 | 96.5 | 97.3 |
| 2 | 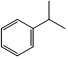 | 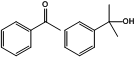  1:2 | 88.5 | 88.3 |
| 3 | 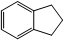 | 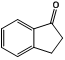 | >99 | 72.9 |
| 4 | 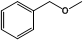 | 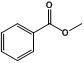 | >99 | 65.4 |
| 5 | 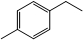 | 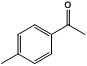 | 95.5 | 89.6 |
| 6 | 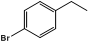 | 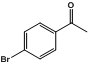 | >99 | 96.5 |
| 7 | 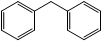 | 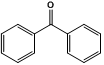 | >99 | 97.8 |
| 8 | 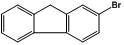 | 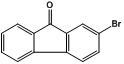 | 99.1 | >99 |
| 9 | 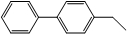 | 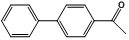 | >99 | 99.5 |
| 10 | 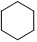 | 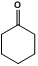 | 51.3 | 66.4 |
| *Reaction conditions: substrate (1.0 mmol), TBHP (3.5 mmol, 70 wt% in water), catalyst (15 mg), H_2_O (3 mL), 80 ^o^C, 12 h; the conversion was determined by GC.* | | | | |

| **Table S2 \|** Catalytic performance of the catalysts for ethylbenzene oxidation. | | | |
| --- | --- | --- | --- |
| 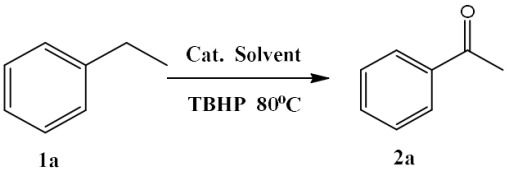 | | | |
| Entry | *Catalyst* | *Conv./%* | *Sel./%* |
| 1 | M-Co-N-C-9^a^ | 96.5 | 97.3 |
| 2 | LC-N-3.6^b^ | 95.4 | 84.4 |
| 3 | S-N-C-900^c^ | 95.3 | 94.1 |
| 4 | A@MMO-0.25^d^ | 92.8 | 89.4 |
| 5 | 6/Mn-MCM-41^e^ | 57.7 | 82 |
| 6 | LDH/G-0.4^f^ | 96.8 | 95.4 |
| *^a^ Reaction conditions: substrate (1.0 mmol), TBHP (3.5 mmol, 70 wt% in water), catalyst (15 mg), H2O (3 mL), 80 ^o^C, 12 h; the conversion and yield were determined by GC.*  *^b^ Ref. 1: Reaction conditions: substrate (1.0 mmol), TBHP (3.0 mmol, 65 wt% in water), catalyst (0.01 g), H_2_O (3 mL), 80 ^o^C, 24 h.*  *^c^ Ref. 2: Reaction conditions: substrate (1.0 mmol), TBHP (3.5 mmol, 70 wt% in water), catalyst (15 mg), H_2_O (3 mL), 80 ^o^C, 12 h.*  *^d^ Ref. 3: Reaction conditions: substrate (20mmol), TBHP (20 mmol), catalyst (0.05 g), acetonitrile 10ml (50 vol.%), 80 ^o^C, 6h.*  *^e^ Ref. 4: Reaction conditions: substrate (10 mmol), TBHP (30 mmol), catalyst (100 mg), 120 ^o^C, 12 h.*  *^f^ Ref. 5: Reaction conditions: substrate (10 mmol), TBHP (40 mmol), catalyst (100 mg), 120 ^o^C, 12h.* | | | |

# Reference

1. Gao, Y., Hu, G., Zhong, J., Shi, Z., Zhu, Y., Su, D.S., Wang, J., Bao, X., Ma, D. (2013) Nitrogen-Doped sp2-Hybridized Carbon as a Superior Catalyst for Selective Oxidation. *Angew. Chem. Int. Ed.* 52, 2109-2113. doi: 10.1002/anie.201207918
2. Li, K., Jie, S. S., Li, Y., Lin, X., Liu, G. Z. The synthesis of N, S-co-doped ordered mesoporous carbon as an efficient metal-free catalyst for selective oxidation of arylalkanes. *Catal. Commun.* 112 (2018) 39-42. doi: 10.1016/j.catcom.2018.04.012
3. Xie, R. F., Fan, G., Yang, L., Li, F. (2016). Hierarchical flower-like Co-Cu mixed metal oxide microspheres as highly efficient catalysts for selective oxidation of ethylbenzene. *Chem. Eng. J.* 288, 169-178. doi: 10.1016/j.cej.2015.12.004
4. Parida, K. M., Dash, S. S., (2009). Manganese containing MCM-41: Synthesis, characterization and catalytic activity in the oxidation of ethylbenzene. *J. Mol.Catal. A: Chem.* 306, 54-61. doi: 10.1016/j.molcata.2009.02.022
5. Xie, R. F., Fan, G., Yang, L., Li, F. (2016). Highly Efficient Hybrid Cobalt–Copper–Aluminum Layered Double Hydroxide/Graphene Nanocomposites as Catalysts for the Oxidation of Alkylaromatics. *ChemCatChem.* 8, 363-371. doi: 10.1002/cctc.201500890
